# Quantifying Circulating IgY Antibody Responses against Select Opportunistic Bacterial Pathogens and Correlations with Body Condition Factors in Wild American Alligators, *Alligator mississippiensis*

**DOI:** 10.3390/biology11020269

**Published:** 2022-02-09

**Authors:** Bailey M. Alston, Thomas R. Rainwater, Benjamin B. Parrott, Philip M. Wilkinson, John A. Bowden, Charles D. Rice

**Affiliations:** 1 Department of Biological Sciences, Clemson University, Clemson, SC 29634, USA; bmalsto@g.clemson.edu; 2 Tom Yawkey Wildlife Center, Georgetown, SC 29440, USA; trrainwater@gmail.com (T.R.R.); Philmwilk@gmail.com (P.M.W.); 3 Belle W. Baruch Institute of Coastal Ecology and Forest Science, Clemson University, Georgetown, SC 29440, USA; 4 Savannah River Ecology Laboratory, Odum School of Ecology, University of Georgia, Jackson, SC 29831, USA; benparrott@srel.uga.edu; 5 Center for Environmental and Human Toxicology, Department of Physiological Sciences, College of Veterinary Medicine, University of Florida, Gainesville, FL 32610, USA; john.bowden@ufl.edu

**Keywords:** American alligator, IgY, mAb AMY-9, disease ecology, immune responses, environmental immunology

## Abstract

**Simple Summary:**

Immunoglobulin Y (IgY) was purified from American alligator, *Alligator mississippiensis*, serum and used to develop a monoclonal antibody (mAb AMY-9) specific for the heavy chains of IgY. This antibody tool was then used to develop an ELISA for quantifying serum antibody responses against whole bacterial pathogens in alligators sampled in Florida, USA and South Carolina, USA. Antibody responses against some of the bacteria were very robust and varied by location and year, and in general these antibody responses correlated well with body condition factors, such as body-mass-indices (BMI). A novel mAb is now available to the scientific community interested in disease ecology of alligators.

**Abstract:**

Little is known about the disease ecology of American alligators (*Alligator mississippiensis*), and especially how they respond immunologically to emerging infectious diseases and zoonotic pathogens. In this study, we examined serum samples collected from wild alligators in Florida (2010–2011) and South Carolina (2011–2012, 2014–2017) for antibody responses to multiple bacteria. Immunoglobulin Y (IgY) was purified from serum to generate a mouse monoclonal antibody (mAb AMY-9) specific to the IgY heavy chain. An indirect ELISA was then developed for quantifying antibody responses against whole cell *Escherichia coli,* *Vibrio parahaemolyticus*, *Vibrio vulnificus, Mycobacterium fortuitum, Erysipelothrix rhusiopthiae*, and *Streptococcus agalactiae*. In Florida samples the primary differences in antibody levels were between January–March and late spring through summer and early fall (May-October), most likely reflecting seasonal influences in immune responses. Of note, differences over the months in antibody responses were confined to *M. fortuitum*, *E. rhusiopthiae*, *V. vulnificus*, and *E. coli*. Robust antibody responses in SC samples were observed in 2011, 2014, and 2015 against each bacterium except *E. coli*. All antibody responses were low in 2016 and 2017. Some of the highest antibody responses were against *V. parahaemolyticus*, *M. fortuitum*, and *E. rhusiopthiae.* One SC alligator estimated to be 70+ years old exhibited the highest measured antibody response against *V. parahaemolyticus* and *M. fortuitum.* By combining data from both sites, we show a clear correlation between body-mass-indices (BMI) and antibody titers in all six of the bacteria examined. Our study provides a critical antibody reagent and a proof-of-concept approach for studying the disease ecology of alligators in both the wild and in captivity.

## 1. Introduction

The American alligator, *Alligator mississippiensis*, is a crocodilian distributed throughout the Southeastern USA, from Texas to North Carolina [1]. Due to heavy and largely unregulated hunting pressure from the 1870s to the 1960s, these crocodilians dwindled in numbers until they were protected under the Endangered Species Conservation Act of 1969 (a precursor to the Endangered Species Act of 1973), and thereafter removed from the list in 1987 as populations recovered [2,3]. Today, although alligators are categorized as a species of Least Concern by the International Union for Conservation of Nature [4], populations remain vulnerable to multiple conservation threats including habitat degradation, pollution, invasive species, and climate change [5]. Reproductive traits of alligators are key factors in the ability of these animals to withstand environmental stressors, and healthy habitat and adaptability to changing environmental factors are essential to reproductive success [6,7,8]. However, the crocodilian immune system is also impacted by environmental stressors (e.g., pathogens), and a healthy immune system is a key measure of fitness in terms of ecological and evolutionary success [9,10,11].

Compared to other vertebrates, the link between basic immunobiology and effects of environmental stressors on immune function in reptiles is not well understood, though this field is rapidly advancing [12,13]. While most aspects of innate immunity, including biochemical and cellular components are conserved across all vertebrates, much less is known about pathogen-specific immune responses in reptiles [14,15], particularly crocodilians. Only a small number of studies have examined the effects of environmental contaminants on crocodilian immune function, and even fewer have assessed the immunological effects of bacterial pathogens on these animals. Thus, there is a clear need for advancement in our understanding of immunological responses of free-ranging crocodilians to naturally occurring pathogens within their habitats.

A key factor in understanding and possibly managing the pathogenic component of environmental stressors is monitoring the presence of various pathogens, and especially those deemed to be opportunistic [16]. Monitoring the prevalence and extent of pathogenic outbreaks is easily performed by PCR using environmental samples [17,18,19]. However, this information may not reveal the potential for infectivity, transmission, or immunological responses over time and distance [16]. Reptiles produce immunoglobulin M (IgM) early in immune responses, and later produce IgY as their mature antibody response to immunogens after isotype switching [20]. Importantly, the drawback of quantifying IgM responses is that serum IgM also contains natural antibodies against a variety of carbohydrates, glycosylated proteins, and pathogen associated molecular patterns, leading to a high degree of cross-reactivity. Therefore, the ability to measure pathogen specific IgY responses would enable investigators to determine what types of pathogens crocodilians (and other reptiles) respond to immunologically with a high degree of specificity. Further, studies that incorporate large numbers of samples collected from different localities over several years would be particularly useful for examining spatial and temporal patterns of crocodilian exposure and immunological response to naturally occurring bacterial pathogens.

The first objective of this study was to purify alligator IgY using standard methods and to generate a high-quality monoclonal antibody (mAb) specific to a single epitope of the IgY protein. Monoclonal antibodies are secreted by hybridoma cell lines, providing an endless supply of the same antibody, which would then allow us and others to quantify alligator responses against a variety of possible environmental pathogens that are known to impact crocodilians [21]. As such, the second study objective was to use the alligator IgY-specific mAb to examine the response of wild alligators to multiple opportunistic pathogens over time and geographical distribution. As a proof of concept, we herein utilized frozen and archived alligator serum samples collected from sites in Florida (FL) and South Carolina (SC), USA. By using archived individual serum samples and body indices data we promote efforts to reduce, replace, and refine studies involving wildlife [22]. 

We show that alligators respond to select environmental pathogens, and these responses may vary from year to year, month to month, and geographic distribution. Moreover, we show that antibody responses correlate well with body condition factors. Ultimately, we provide a novel tool for biologists studying disease ecology and environmental biology of alligators, as well as demonstrate a proof of concept for future monitoring studies. 

## 2. Materials and Methods

### 2.1. Alligators and Serum Collections

The study described herein utilized archived samples collected from historical studies on alligator reproduction and genetics. Blood samples were obtained from a total of 35 female and 76 male adult alligators from Merritt Island National Wildlife Refuge, Florida (28°38′28″ N, 80°44′08″ W) at monthly intervals from January 2010 through January 2011, and from a total of 40 female and 30 male adult alligators at various times during 2011, 2012, 2014, 2015, 2016, and 2017 at the Tom Yawkey Wildlife Center (33°12′33″ N, 79°17′46″ W), South Carolina. Immediately following capture, blood was collected from each animal from the post-occipital sinus of the spinal vein [23] using a sterile needle and syringe [24,25,26]. Whole-blood samples were then transferred to 8- or 10-mL lithium–heparin Vacutainer blood-collection tubes (BD), stored on ice in the field, and later centrifuged at 2500 rpm at 4 °C for 10 min to obtain serum, which was stored at –80 °C until analysis [27]. Sex (M, F), snout-vent length (SVL), and tail girth (TG) were recorded for each animal [28], and each blood sample was labeled with a unique identification number. Body mass indices (BMI) for individuals were calculated as previously described using the formula BMI = (TG/(SVL × 2) [26]. Following sample and measurement collection, each animal was tagged and released at its site of capture. 

### 2.2. IgY Purification 

Previous work demonstrated that IgY can be purified from reptile serum using the immunoglobulin-binding protein-G from Streptococcal bacteria [29,30]. To purify IgY from alligator serum in this study, a Protein-G column (Thermo Fisher) was equilibrated by running 10 mL of 0.10 M Tris pH 8.0 through the columns followed by 10 mL of 0.01 M Tris pH 8.0. A pooled serum composite in a 15 mL tube was generated using 100 µL from 60 random alligator samples. The tube then received 600 µL 1 M Tris, pH 8.0. The pooled serum sample was centrifuged for 10 min at 1000× *g* to remove aggregates and passed twice through the column. Twenty mL of 0.10 M Tris pH 8.0, and then 20 mL 0.01 M Tris pH 8.0 were subsequently passed through the column. Finally, IgY was eluted with 0.05 M glycine at pH 2.5 as 1 mL aliquots with 200 µL of 1.0 M Tris pH 8.0 added to each. Eluted fractions from Protein-G columns were pooled according to fractions containing proteins as determined using the BCA protein assay (Thermo Fisher). To confirm that the collected product was pure and of expected size and composition, samples were subjected to SDS-PAGE under reducing and denaturing conditions using 5× Laemmli sample buffer containing 2-mercaptoethanol (2-ME) and boiled for 8 min. Samples were then pipetted to 4–20% Mini-PROTEAN^®^ TGX Stain-Free™ Protein Gels (BioRad, Richmond CA, USA). BioRad precision pre-stained protein molecular weight markers or BLUelf™ (FroggaBio, Buffalo NY, USA) pre-stained markers were used as standards. Electrophoresis was conducted at 180 V and then gels were stained with Coomassie blue for 16 h, followed by de-staining with a 30% methanol/10% acetic acid solution. The gel was then framed and dried using a framing apparatus (Diversified Biotech, Dedham MA, USA) and imaged.

### 2.3. Generation of Monoclonal Antibody against IgY 

To generate alligator IgY specific monoclonal antibody, 6-week-old female Balb/c mice were immunized subcutaneously (s.c.) with 100 µg of purified IgY in a volume of 100 µL 0.01 M, pH 7.2 phosphate buffered saline (PBS) mixed with 100 µL TiterMax^®^ Gold (Atlanta, GA USA) adjuvant. Fourteen days later, mice were immunized again s.c. using Freud’s incomplete adjuvant, and again on day 35 without adjuvant. On day 56 mice were immunized via intraperitoneal injections with 50 µg IgY. Five days later, mice were euthanized by slow lethal CO_2_ asphyxiation, followed by bilateral pneumothorax according to an approved Clemson University IACUC Animal Use Protocol, blood collected by cardio-puncture, and spleens harvested to isolate plasma cells. Blood was clotted overnight at 4 °C, then centrifuged for 20 min at 10,000× *g* to collect overlying serum as the crude source of polyclonal anti-sera if needed and stored at −20 °C. Procedures for fusion of spleen cells with Sp2/0–Ag14 myelomas have been described elsewhere [31,32]. Primary hybridomas were typically grown in Dulbecco’s Modified Eagle Medium (Cellgro) supplemented with heat inactivated fetal bovine serum (FBS, Gibco), 20 mM HEPES, 10 mM L-glutamine, 100 µg/mL penicillin, 100 µg/mL streptomycin, 110 µg/mL sodium pyruvate, 1% non-essential amino acids (100× stock), 4.5 g/L glucose, 10 µg/mL gentamycin and 5 µg/mL nystatin.

Primary hybridomas were first screened against purified IgY by Enzyme Linked Immunosorbent Assay (ELISA) and cells from positive wells were cloned by limiting dilution using our previously described approaches [30,32,33]. Monoclonal hybridomas were grown to confluence, and the supernatants collected by centrifugation, then treated with 0.05% NaN_3_ and stored at 4°C. Isotyping the antibody within hybridoma supernatants was carried out using Pierce Rapid Antibody Isotyping Kits for mouse (Thermo Fisher). Finally, to determine the Ig chain specificity of mAbs for purified IgY and whole serum IgY, samples were first subjected to SDS-PAGE as described above, then transferred to two BioRad Immun-Blot^®^ PVDF (polyvinylidene fluoride) membranes at 100 V for 60 min on ice. Upon transfer, these blots were blocked at 4 °C for 16 h with 10% fetal bovine serum in PBS. Next, the blots were probed for 1 h at room temperature with hybridoma supernatants diluted 1:3 in complete cell culture media. Blots were then washed 3× for 5 min with PBS containing 0.05% tween-20 detergent (PBS-TW20). The blots were then probed with goat anti-mouse IgG conjugated with alkaline phosphatase (ThermoFisher, 1:1000) for one hour at room temperature, followed by 3 × 5 min wash steps with PBS-TW20. Finally, alkaline phosphatase activity was visualized using the substrate Nitro Blue Tetrazolium (NBT) and 5-bromo-4-chloro-3’-indolyphosphate (BCIP) in alkaline phosphatase (AP) buffer (100 mM NaCl, 5 mM MgCl_2_, 100 mM Tris, pH 9.5). Subsequent steps demonstrated that mAb AMY-9, an IgG_1_ *κ* immunoglobulin, is most useful for all subsequent assays. 

### 2.4. Development of ELISAs against Opportunistic Aquatic Bacteria 

In this study, *Escherichia coli,* *Vibrio cholera, Vibrio parahaemolyticus, Vibrio anguillarum, Vibrio vulnificus, Brevundimonas vesicularis, Mycobacterium fortuitum,* and *Erysipelothrix rhusiopthiae* were obtained from ATCC (Manassas, VA, USA), and grown using recommended materials and methods. *Streptococcus agalactiae* cultures were a generous gift from Dr. John Hawke, Louisiana State University, and grown as directed. High bonding 96 well plates (Corning, #9018) were coated for 16 h at 4 °C with 75 µL of a 1 mg mL^−1^ solution of poly-D-lysine (VWR Scientific) in distilled water, followed by wash steps with phosphate buffered saline containing 0.05% tween-20 (PBS-TW20). To perform ELISAs against whole cells cultures adhered to ELISA plates, cultures were first grown under conditions as previously described [33]. Optimized suspensions of live bacterium were then prepared and 50 µL of bacterium were added to wells using our lab’s modifications [33,34,35] of the original assay [36], including fixation and blocking steps. To initiate assays, serum suspensions were made by adding 10 µL of serum from each individual animal into 990 µL of PBS-TW20 in 1.5 mL snap cap tubes to yield a dilution at 1:100. As a reference sample, a composite sample for each species comprised of serum from randomly selected individuals in PBS was made at a dilution of 1:100. Eighty µL of each diluted sample, including the composite reference sample, were added in duplicate to the respective wells. Each plate also contained two wells of 80 µL PBS-TW20 as an assay control. The plates were incubated overnight at 4 °C and washed 4 times with PBS-TW. As the source of primary antibody, AMY-9 hybridoma supernatant from a single-batch confluent culture was diluted 1:3 with hybridoma media and 80 µL added to all wells of the plate and incubated at room temperature for 2 h, followed by fours washes in PBS-TW. The remainder of steps were routine and described elsewhere (Rodgers et al., 2018). Goat anti-mouse IgG AP (1:1000) was added in 75 µL volumes to each well and incubated at room temperature for 1.5 h before being washed four times with PBS-TW20. Finally, 80 µL of 1 mg/mL p-nitrophenol phosphate (ThermoFisher) in AP buffer were added to each well, the plates were incubated for 30 min at room temperature, and the reaction was stopped with 100 ul 2 M NaOH. Optical densities for all plates were read at 405 nm and the data recorded. Relative antibody titers were calculated as the optical density (OD) multiplied by the serum dilution factor of 1:200 [30,33,35].

### 2.5. Statistical Analysis

The values for serum titers against bacteria, calculated as relative OD at a 1:200 dilutions, from individual alligators were organized into categories based on state of collection (SC or FL), month or year of collection, sex, SVL, TG, and BMI. Initial analysis showed no differences between males and females for all types of measurements (*p* ≥ 0.05), and thereafter data from both sexes were pooled. Because only two serum samples were collected in 2012, these data were not included in year-to-year statistical analysis but were included during comparisons of all SC samples. Tail girth data were not collected for one SC alligator and one FL alligator; therefore, data from these two animals were not used in BMI calculations and were excluded in correlation analysis between BMI and antibody titers. Antibody titers in SC alligators against each bacterial pathogen were compared by year using a non-parametric ANOVA (Kruskal–Wallis) analysis with a Dunn’s multiple contrast post-hoc test whenever differences were noted by the ANOVA and appropriate for the analysis. Antibody titers in FL alligators were compared by month of collection using the same statistical approach. Correlations between body size (SVL) and condition factor (TG, BMI) and antibody titers were determined by Pearson’s correlation coefficient using GraphPad Prism9′s statistical software. The α-value was set at 0.05 prior to the study.

## 3. Results

### 3.1. Serum Immunoglobulin Y Purification and Reactivity of mAb AMY-9

**Figure 1:**
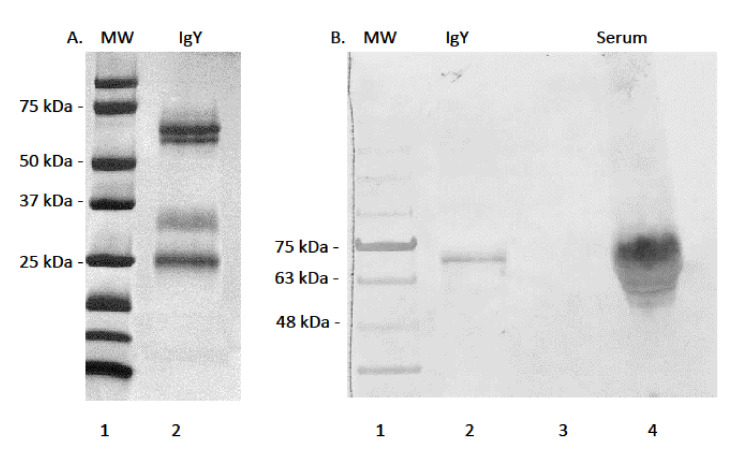
(**A**). SDS-PAGE and Coomassie blue staining of Protein-G purified alligator IgY. Lane 1: molecular weight marker; Lane 2: alligator IgY. Strong bands appear at approximately 63 kDa, 34 kDa, and 27 kDa, corresponding to the heavy chains, a truncated form, and the light chains of IgY, respectively. (**B**). Immuno-reactivity of mouse mAb AMY-9 against purified alligator IgY and whole serum. Lane 1: molecular weight marker; lane 2: purified IgY; lane 3 blank: lane 4; pooled serum. mAb AMY-9 is specific for the heavy chain of IgY.

Efforts to purify alligator IgY using methods previously employed for sea turtle IgY purification were successful in that very pure samples were eluted from columns (Figure 1A). Strong banding of proteins under denaturing and reducing conditions was observed by SDS-PAGE, revealing a heavy chain of approximately 65 kDa, corresponding to the heavy chain of typical full length IgY. The IgY heavy chain eluted as a slight double band, corresponding to either degraded heavy chain under reducing conditions, or because of asymmetry in IgY as reported for green sea turtles [29]. Purified alligator IgY also contained a protein of approximately 34 kDa corresponding to the truncated isoform of IgY [37]. A smaller band of approximately 27 kDa corresponding to the light chains of typical full length and truncated IgY was eluted. Efforts to develop and characterize a mAb against alligator IgY were successful in that mAb AMY-9 reacted against the 65 kDa heavy chain of IgY in both purified and whole serum samples, indicating high specificity for epitopes located on the heavy chain not shared with the truncated form and light chain (Figure 1B).

### 3.2. Antibody Titers and Condition Factors

**Figure 2:**
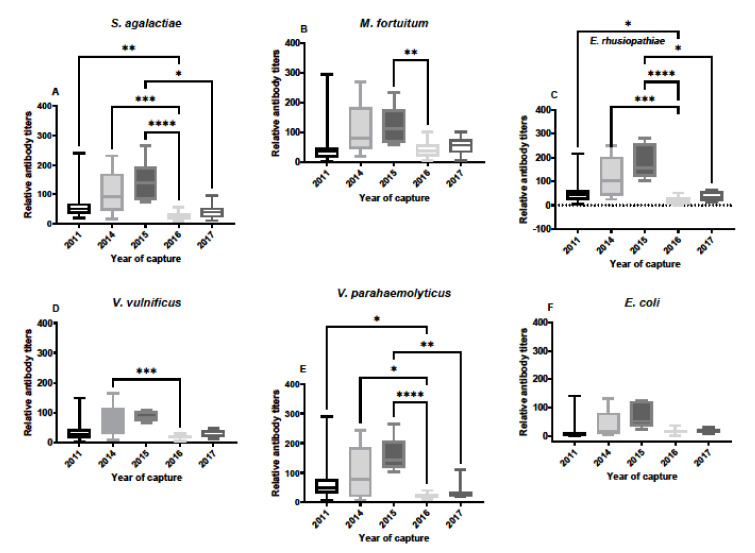
Relative antibody titers against bacterial pathogens *S. agalactiae* (**A**), *M. fortuitum* (**B**), *E. rhusiopthiae* (**C**), *V. vulnificus* (**D**), *V. parahaemolyticus* (**E**), and *E. coli* (**F**) by year in South Carolina alligator serum samples. Sample sizes by year were N = 23 for 2011, N = 12 for 2014, N = 8 for 2015, N = 19 for 2016, and N = 8 for 2017. Titers against all bacteria were lower in 2011 and 2017 for each of the bacteria. Titers against *E. coli* did not differ over the years of capture. Data are represented as box plots showing median titer, the upper and lower first quantile, and the bars represent the ranges of titer data. (* denotes *p* ≤ 0.05, ** denotes *p* ≤ 0.01, *** denotes *p* ≤ 0.001, **** denotes *p* ≤ 0.0001).

Antibody titers in SC serum samples varied over the years of sample availability, and between each of the different bacteria (Figure 2). One of the key observations was that some of the serum samples contained much higher titers against bacterial pathogens than the group for each year. For example, in 2011 antibody titers were very high in a few animals in comparison to most animals. Additionally, important is that the responses from year to year were not the same for each bacterium, indicating that responses to one bacterium were not linked to the others, which is often the case with IgM antibodies, and the main reason for exploring IgY responses in this study. In general, responses against bacterial pathogens were higher in 2014 and 2015, with low responses in 2011, 2016 and 2017. Moreover, antibody responses to *E. coli* were very low in these serum samples, especially in comparison to *S. agalactiae* and *E. rhusiopthiae*.

**Figure 3:**
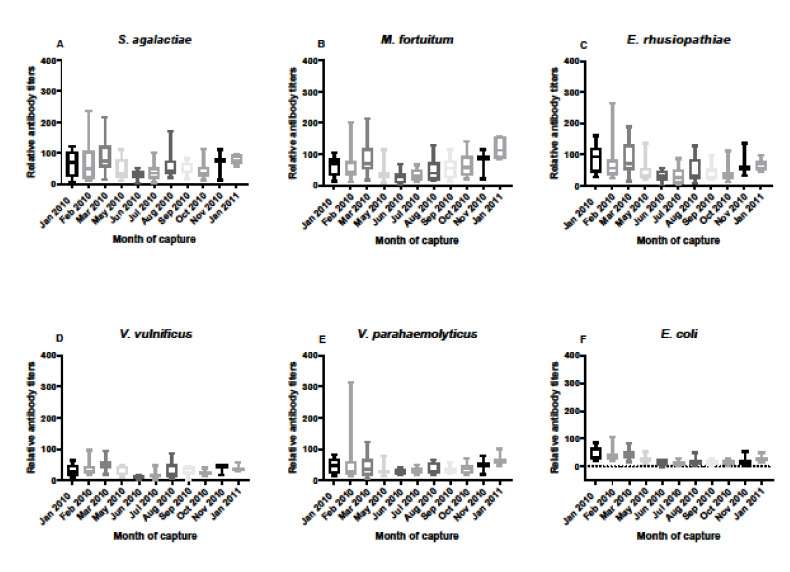
Relative antibody titers against bacterial pathogens *S. agalactiae* (**A**), *M. fortuitum* (**B**), *E. rhusiopthiae* (**C**), *V. vulnificus* (**D**), *V. parahaemolyticus* (**E**), and *E. coli* (**F**) by month from January 2010 through January 2011 in Florida alligator serum samples. Sample sizes by month were N = 10 for January, N = 15 for February, N = 13 for March, N = 11 for May, N = 7 for June, N = 15 for July, N = 13 for August, N = 10 for September, N = 9 for October, N = 3 for November, and N = 7 for January 2011. Differences in relative titers by month differed only in *M. fortuitum*, *E. rhusiopthiae*, and *V. vulnificus*, and *E. coli*. Data are represented as box plots showing median titer, the upper and lower first quantile, and the bars represent the ranges of titer data. Statistical differences by month and bacterium are provided in Table 1.

Antibody responses in serum samples from FL alligators varied throughout the year of sampling from January 2010 through January 2011 (Figure 3). While many statistical differences were revealed from the multi-comparisons, the primary differences were between early periods of the year (January–March) and late spring through summer and early fall (May–October), most likely reflecting seasonal influences in immune responses (Table 1). Of note, differences over the months in antibody responses were confined to *M. fortuitum*, *E. rhusiopthiae*, *V. vulnificus*, and *E. coli*. Responses to *S. agalactiae* and *V. parahaemolyticus* were not associated with seasonality or other influences.

**Figure 4:**
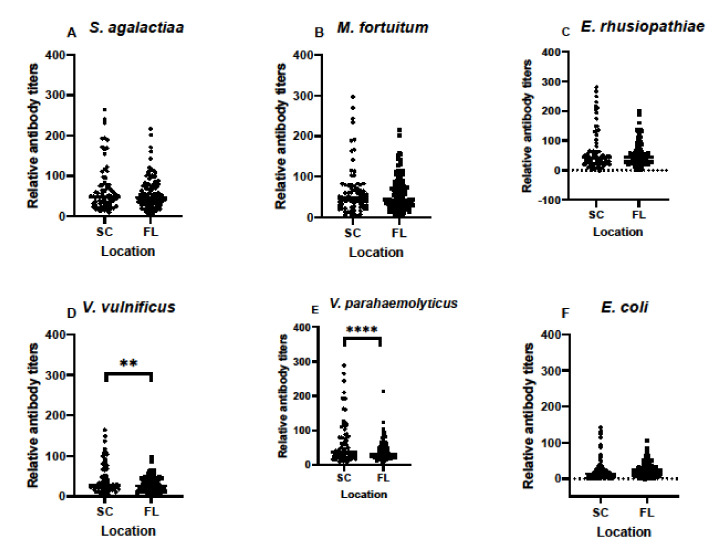
Relative antibody titers against bacterial pathogens *S. agalactiae* (**A**), *M. fortuitum* (**B**), *E. rhusiopthiae* (**C**), *V. vulnificus* (**D**), *V. parahaemolyticus* (**E**), and *E. coli* (**F**) in all South Carolina alligator serum samples compared to all Florida alligator serum samples. Total sample size for this comparison was N = 73 for South Carolina samples and N = 112 for Florida samples. Data are represented in scatter plots to show the distribution of antibody titers in all available serum samples. Differences in titers between locations were noted for *V. vulnificus* and *V. parahaemolyticus*, but not the other four species of bacteria. (** denotes *p* ≤ 0.01, **** denotes *p* ≤ 0.0001).

An important part of any monitoring program to assess immune functions in animals throughout their ranges is to compare data from multiple sites when possible. In our case, we wanted to determine if differences existed in archived samples from SC and FL, regardless of time of year or year of sampling. Based on serum samples alone, and without considering other factors, antibody titers in samples from SC and FL differed only in responses to *V. vulnificus* and *V. parahaemolyticus* (Figure 4). Compared to responses against the other four bacteria examined, those against *E. coli* were the lowest in both SC and FL.

**Figure 5:**
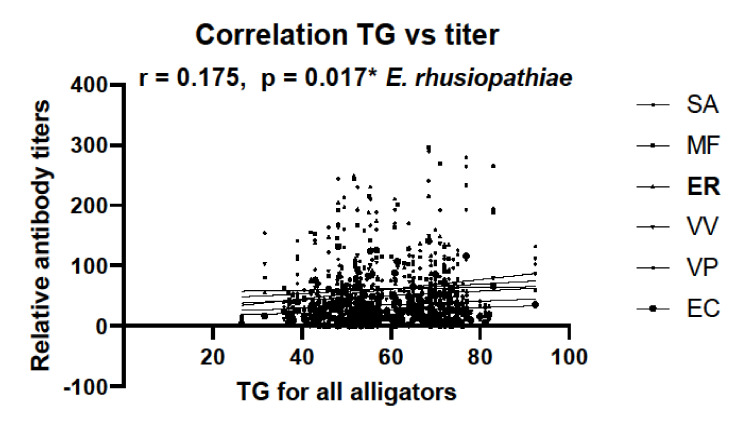
Correlation between tail girth (TG) and relative antibody titers against all six bacteria calculated from all available TG data with all alligator serum samples. Combined sample size was N = 183. Pearman’s correlation coefficient is shown (r = 0.175), and only antibody titers against *E. rhusiopthiae* correlated with TG (* denotes *p* = 0.017).

Measures of SVL and TG for each animal from which serum was collected were used to determine any correlation with antibody titers. When comparing animals from SC and FL, no correlation between SVL or TG and antibody titers was observed (data not shown). However, when antibody titer data from all samples were combined, a correlation was observed between TG and responses against *E. rhusiopthiae*, but not other bacterium examined (Figure 5).

**Figure 6:**
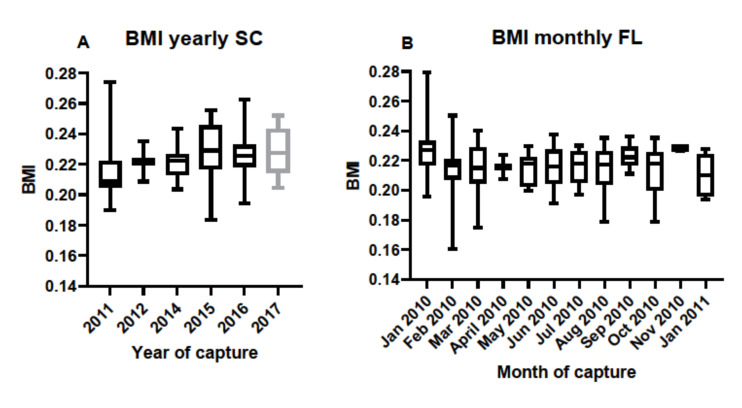
(**A**) Calculated body mass indices (BMI) in South Carolina alligators corresponding to individual serum samples collected over the period between 2011 and 2017. Sample sizes were N = 23 in 2011, N = 2 in 2012, N = 12 in 2014, N = 8 in 2015, N = 19 in 2016, and N = 8 in 2017. There were no differences in BMI over the years in South Carolina alligators. (**B**). Calculated BMI in Florida alligators corresponding to individual serum samples collected on a monthly basis between January 2010 and continuing through January 2011. Sample sizes were N = 11 for January, N = 13 for February, N = 11 for March, N = 2 for April, N = 10 for May, N = 6 for June, N = 14 for July, N = 14 for August, N = 10 for September, N = 9 for September, N = 9 for October, N = 3 for November, and N = 8 for January 2011. There were no differences in Florida alligator BMI during the monthly sampling for this period.

**Figure 7:**
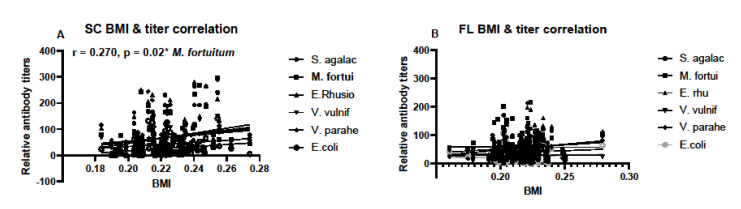
Correlation between calculated body mass indices (BMI) and relative antibody titers in South Carolina (**A**) and Florida (**B**) alligators and corresponding individual serum samples. Data from all South Carolina and all Florida alligators were used to determine correlation. Sample sizes were N = 72 for South Carolina and N = 111 for Florida. Only relative antibody titers against *M. fortuitum* and BMI were found and only in South Carolina samples (r = 0.270, * denotes *p* = 0.02).

**Figure 8:**
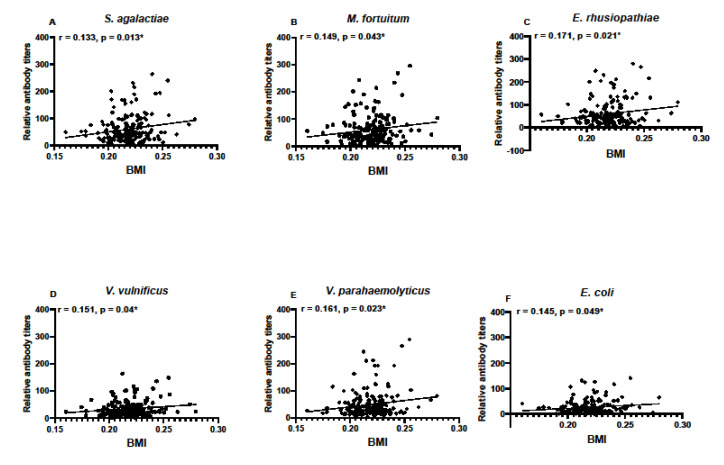
Correlation between calculated body mass indices (BMI) and relative titers against bacterial pathogens *S. agalactiae* (**A**), *M. fortuitum* (**B**), *E. rhusiopthiae* (**C**), *V. vulnificus* (**D**), *V. parahaemolyticus* (**E**), and *E. coli* (**F**) when BMI data and antibody titer data were pooled for all alligators in the study (n = 183). Relative antibody titers against all six bacteria positively correlated with BMI (* denotes *p* ≤ 0.05). Pearman’s correlation coefficients are noted for each bacterium examined.

Body mass index was calculated for individuals based on SVL and TG data and used to determine if condition factors differed between sampling locations. There were no differences in BMI between the years of sampling in SC samples, nor between monthly samples taken in FL (Figure 6). Condition factors in the form of BMI data were then used to determine any correlation between BMI and antibody titers. BMI data correlated with antibody responses to *M. fortuitum*, but not the other bacteria in SC samples (Figure 7A). There was no correlation between BMI and antibody titers in FL samples (Figure 7B). Combining data from both SC and FL to determine if there was a correlation between alligator BMI and antibody titers greatly increased the sample size, leading to a more robust statistical analysis. After doing so, a clear correlation between BMI and antibody titers in all six of the bacteria examined was observed (Figure 8). 

## 4. Discussion

In this study we demonstrate that alligator IgY is easily purified from serum using Protein-G, which is consistent with sea turtles, and possibly other reptiles as well. This contrasts with avian IgY, which cannot be purified by Protein-G or protein-A, at least from egg-yolk which is the most common source for IgY purification in birds. To our knowledge, IgY from reptilian eggs using Protein-G has not been published but considering the need to protect eggs of threatened and endangered sea turtles and crocodilians for conservation purposes this may not be a viable option. This study also generated an alligator IgY-specific monoclonal antibody (mAb AMY-9) that can be used for multiple applications related to conservation efforts, disease ecology, and animal husbandry. Mouse monoclonal antibodies are preferred over rabbit polyclonal antibodies because they are secreted by immortalized hybridomas in vitro, and thus there would be no need to immunize mice or rabbits in the future. Furthermore, monoclonal antibodies are highly specific to each’s epitope. Moreover, generating additional antibodies for anyone interested in the antibody for additional studies would simply require growing more hybridomas in vitro to collect supernatants prior to antibody purification. 

Wildlife populations are continuously vulnerable to pathogen outbreaks due to viruses, bacteria, fungi, and parasites that naturally occur in those populations or due to emerging infectious diseases. Monitoring the prevalence and extent of these outbreaks is easily done by PCR using water, soil, or tissue samples [17,18,19]. For example, farmed alligators are known to be an environmental reservoir for West Nile virus [38]; however, the extent to which alligators immunologically respond to the virus with specific IgY production remains unknown. Also, while there are examples of other crocodilians being sero-positive for the human pathogen *Leptospira*, and suggestions that humans may acquire *Leptospira* from crocodilians [39,40,41]. we are unaware of documented zoonotic disease transmission between humans and American alligators. Immunoglobulin Y is closely related to IgG and IgE of higher vertebrates, and several immunoglobulin subclasses function to neutralize viruses. Future studies will focus on viral pathogens, but herein we demonstrate that circulating alligator IgY is specific for various bacterial proteins, and antibody levels against whole bacteria protein antigens are robust and may vary by location, year of sampling, time of year of sampling, and relative BMI. Along with PCR-aided environmental monitoring for possible alligator pathogens of interest, we can now test for sero-positivity in alligators using mAb AMY-9. 

This study ultimately focused on six of the myriads of potential aquatic pathogens known to affect multiple species. Two of these, *V. vulnificus* and *V. parahaemolyticus* are of concern to humans because their prevalence, and possibly virulence, has increased with warming water temperatures along coastal Gulf of Mexico and the southern Atlantic Ocean [42,43]. These areas are adjacent to well-known alligator habitats, and our data comparing Florida alligator serum samples to those from South Carolina show differences in relative titers for these two bacteria. Of note, these titers were higher in South Carolina samples, where we also found robust responses against *S. agalactiae* and *E. rhusiopthiae* that differed over the years of serum collection. It is possible that severe weather events influenced year to year antibody titer concentrations, or that these pathogens were in higher than usual abundance over the years of sampling. Moreover, the seasonal fluctuations of certain antibody titers in the FL population may be related to temperature, as immune functions in ectotherms are impacted by temperature.

As global temperatures rise, the ability to monitor the environment for both pathogen prevalence and sero-positivity rate may be important in future studies of organisms displaying temperature dependent sex determination (TSD), including crocodilians [44]. Higher global temperatures may favor the prevalence of emerging pathogens of concern [45]. How this may impact wildlife species, including alligators, is yet unknown and will be the focus of future studies that examine sero-positivity rates using mAb AMY-9. Because reptilian immune responses are temperature sensitive [13], another key question is if immunological functions, such as the ability to respond to pathogens with IgY antibodies, will be more effective. It is now appreciated that external temperatures affect alligator anti-microbial activity against select bacteria, and that testosterone affects these activities [46], but how these interactions affect pathogen specific and total IgY levels remains to be determined. 

Over time, warming global temperatures may also result in a gradual increase in the American alligator’s northern distributional limit in the southeastern U.S. Like alligators currently living at the northern extent of their range, some of these animals may be exposed to extreme cold temperatures during the winter. How these changes will impact alligator immunity and disease susceptibility, or ecosystem transfer of pathogens, should be a point of research going forward. One of the key findings of this study is that given a large sample size, such as the pooling of all South Carolina and Florida samples, antibody titers against all six-bacterium correlated positively with BMI. This observation indicates that robust antibody responses may be predicted based on an increase in alligator body condition. However, these data also indicate a very high individual BMI does not necessarily correlate with the highest antibody titers.

One of the key applications for our mAb AMY-9 is to monitor the success of future vaccination programs in alligators, or other crocodilians. The immunogenicity and efficacy of vaccines is almost always determined by the rate and magnitude of specific antibody production and secretion, and the ability of those stimulated antibodies to neutralize the pathogen under consideration. The recovery of alligator populations by the late 1980s following two decades of legal protection eventually led to sustainable use of alligators through farming and ranching programs [4]. As with any commodity species, growing crocodilians at high densities within confined spaces can lead to disease outbreaks [46]. Many of these bacterial diseases could be treated prophylactically using vaccination protocols, and early success could be monitored by immunodiagnostics as is done for the chicken industry (Munhoz et al., 2014). Keeping disease levels low or absent in farmed alligators and other crocodilians would also reduce the chance of spill-over outbreaks in wild populations. 

It is possible that mAb AMY-9 recognizes IgY in a variety of crocodilians. For example, our mAb against loggerhead sea turtle (*Caretta caretta*) IgY heavy chains reacts with all sea turtles except the leatherback (*Dermochelys coriacea*)*,* and a mAb against loggerhead IgY light chains cross reacts with all sea turtles [30]. Therefore, ongoing work should examine the possibility that mAb AMY-9 developed for this study may recognize IgY in other crocodilians, especially the closely related and critically endangered Chinese Alligator (*Alligator sinensis*). One immediate next step is to address this possibility by determining the cross-reactivity of mAb AMY-9 among serum samples in as many crocodilians as possible.

## 5. Conclusions

To our knowledge, we have developed and characterized the first monoclonal antibody against IgY of the American alligator. This novel tool is available to conservation biologists, disease ecologists, and those in comparative and experimental immunology for a variety of applications. As a proof of concept, we have demonstrated that robust antibody responses to diverse aquatic bacterial pathogens are in circulation from two different wild alligator populations in southeastern U.S., and there are several differences between the two populations. Body mass indices data correlate with antibody levels across populations of alligators, and as is the case with other animals, BMI may be an indicator of general health, though more work is needed to determine the nutritional status of animals when using TG and SVL in determining BMI. As with many wildlife conservation efforts, future vaccination protocols can be developed against a variety of diseased alligators (and potentially other crocodilians) in farmed and wild populations, and one measure of vaccine efficacy is elevated specific antibody titers. These antibody titers can now be quantified using a very specific mAb against alligator IgY.

## Figures and Tables

**Table 1:** Statistical significance summary of relative antibody titers against select aquatic bacteria in all alligators from Merritt Island National Wildlifie Refuge, Florida by month between January 2010 and January 2011 as shown in Figure 4. There were no differences in antibody titers against *V. parahaemolyticus* and *S. agalactiae*. * denotes *p* ≤ 0.05. ** denotes *p* ≤ 0.01. *** denotes *p* ≤ 0.001, **** denotes *p* ≤ 0.0001.

*M. fortuitum*	*E. rhusiopthiae*	*V. vulnificus*	*E. coli*
March–July *	January–July *	February–June *	January–June *
May–January 2011 *		March–June ****	January–July ***
June–January 2011 **		March–July ****	January–August **
July–January 2011 ***		May–June *	January–September *
August–January 2011 *		June–January 2011 *	January–October **
			February–July ***
			February–August *
			February–September *
			February–October **
			March–June *
			March–July ***
			March–August **
			March–September *
			March–October **

## Data Availability

Data is contained within the article.
